# Ectopic Cervical Thymoma with Myasthenia Gravis and Pure Red Cell Aplasia: A Case Report

**DOI:** 10.70352/scrj.cr.25-0818

**Published:** 2026-03-27

**Authors:** Eiji Narusawa, Kai Obayashi, Sayaka Obayashi, Toshiteru Nagashima, Natsuko Kawatani, Tomohiro Yazawa, Ryohei Yoshikawa, Nozomi Matsumura, Ken Shirabe, Seshiru Nakazawa

**Affiliations:** 1 Department of General Surgical Science, Gunma University Graduate School of Medicine, Maebashi, Gunma, Japan; 2 Division of Thoracic Surgery, Gunma Prefectural Cancer Center, Ota, Gunma, Japan; 3 Department of Human Pathology, Gunma University Graduate School of Medicine, Maebashi, Gunma, Japan

**Keywords:** ectopic thymoma, myasthenia gravis, pure red cell aplasia, multiple thymoma

## Abstract

**INTRODUCTION:**

Ectopic cervical thymoma is an extremely rare tumor, particularly when associated with myasthenia gravis and pure red cell aplasia.

**CASE PRESENTATION:**

A 65-year-old female was undergoing treatment for myasthenia gravis and pure red cell aplasia at our hospital. Myasthenia gravis symptoms were controlled with prednisolone, and aplasia was managed using oral cyclosporine A. A gradually increasing cervical mass had been noted previously. Needle biopsy of the mass suggested an ectopic thymoma, and the patient was referred for surgery. Preoperative chest CT revealed a 6.5-cm solid mass within the caudal portion of the left thyroid lobe, which was displacing the trachea to the right. No continuity was noted between the cervical lesion and the thymus. Fluorodeoxyglucose PET showed fluorodeoxyglucose uptake in the mass, with a maximum standardized uptake value of 6.48. No other abnormal uptake was observed, including that in the thymus. The preoperative diagnosis was an intrathyroidal ectopic cervical thymoma associated with myasthenia gravis and pure red cell aplasia. Based on the history of myasthenia gravis, both extended thymectomy and left thyroid lobectomy were performed. The postoperative course was uneventful. Histopathological examination showed that the cervical mass was a type AB thymoma. The examination also revealed an occult type A thymoma (Masaoka stage II) in the thymus.

**CONCLUSIONS:**

We encountered a rare case of type AB ectopic cervical thymoma associated with myasthenia gravis and pure red cell aplasia. Extended thymectomy and cervical tumor resection revealed the presence of an occult type A thymoma in the thymus.

## Abbreviations

ECT
: ectopic cervical thymoma
FDG-PET
: fluorodeoxyglucose PET
MG
: myasthenia gravis
PRCA
: pure red cell aplasia
SUV
: standardized uptake value
TdT
: terminal deoxynucleotidyl transferase

## INTRODUCTION

ECT is an extremely rare tumor.^1)^ Here we describe a rare case of type AB ECT in a patient with MG and PRCA. The patient was also found to have an occult type A thymoma in the thymus.

## CASE PRESENTATION

The patient initially visited a hospital with chief complaints of eyelid ptosis and diplopia. She was diagnosed with MG, and treatment with prednisolone (20 mg/day) and pyridostigmine (180 mg/day) was initiated. As symptoms improved, the prednisolone dose was gradually reduced to 5 mg every other day, and the pyridostigmine dose was reduced to 120 mg/day. At that time, a chest CT was performed; however, no thymoma was detected. Fourteen years after the diagnosis of MG, the patient noticed a cervical mass and visited the previous hospital. At that time, the clinicians opted to follow up on the mass without intervention. Eighteen years after the diagnosis of MG, the patient developed anemia. Further investigations led to the diagnosis of PRCA, for which treatment was initiated. The symptoms of these diseases were controlled using 5 mg of prednisolone on alternate days and 120 mg of pyridostigmine for MG and 150 mg of oral cyclosporine for PRCA. Twenty-four years after the diagnosis of MG, the cervical mass was noted again during a health checkup. The patient had noticed enlargement of the mass, prompting the visit to our hospital.

**Fig. 1:**
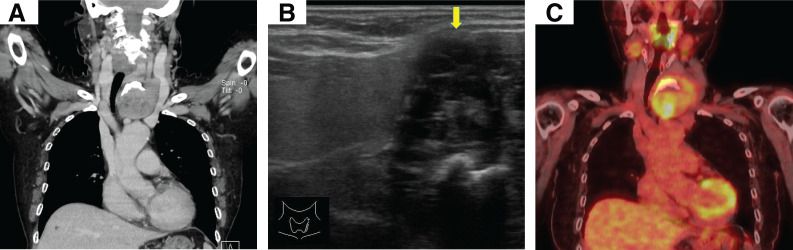
Radiological findings of the tumor. (**A**) Chest CT shows a 6.5-cm mass inferior to the left thyroid lobe, displacing the trachea to the right. (**B**) Ultrasonography showing a heterogeneously hypoechoic mass (yellow arrow) with indistinct margins in the left neck that appeared within the left thyroid lobe. (**C**) Fluorodeoxyglucose PET shows abnormal uptake in the mass.

Blood analysis revealed mild anemia and a reticulocyte count within the normal range. An increased anti–acetylcholine receptor antibody titer (3.7 nmol/L) was observed. Chest CT revealed no mediastinal thymoma but showed a 6.5-cm solid mass abutting the caudal aspect of the left thyroid lobe, accompanied by rightward deviation of the trachea. The cervical lesion showed no continuity with the mediastinal thymus (**Fig. 1A**). Over a 5-year follow-up period, the lesion had increased in size by approximately 5 mm. Ultrasonography suggested that the cervical mass had partial continuity with the thyroid gland, raising the possibility of a thyroid tumor; therefore, a needle biopsy was performed. Histopathological examination revealed a type AB thymoma, and the patient was referred to our department for surgical resection of the ECT (**Fig. 1B**). FDG-PET showed FDG uptake in the cervical mass with a SUV max of 6.48. No other abnormal uptake was present, including the thymus (**Fig. 1C**).

The preoperative diagnosis was ECT, with concomitant MG and PRCA. Given the presence of MG, we planned to resect the ECT and also to perform an extended thymectomy. As the cervical lesion was not contiguous with the thymus, we concluded that removal of ECT was feasible via a cervical approach alone. Accordingly, we planned a left thyroid lobectomy, including resection of the cervical lesion, followed by a video-assisted thoracoscopic extended thymectomy.

Video-assisted thoracoscopic extended thymectomy was performed using 4 ports (**Supplementary Fig. 1**): a 12-mm port was placed in the right sixth intercostal space along the midclavicular line, and two 5-mm ports were inserted in the right fourth and fifth intercostal spaces along the anterior axillary line; and an additional 12-mm port was placed below the xiphoid process.

Intraoperatively, the cervical lesion showed strong adhesion to the thyroid gland, suggesting invasion to the thyroid, but showed no invasion into the other surrounding tissues. The lesion was bluntly dissected and excised. The postoperative course was uneventful, and the patient was discharged 7 days after the surgery.

**Fig. 2:**
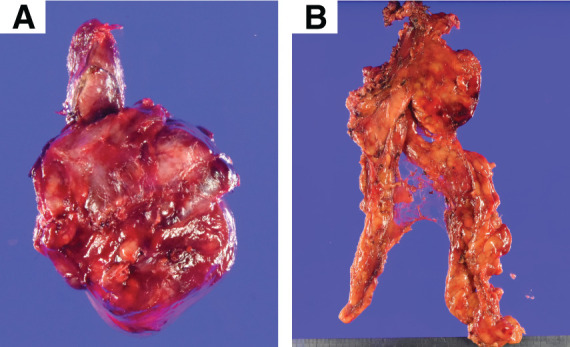
Postoperative specimens—gross findings. Resected specimen of the ectopic cervical thymoma and left thyroid lobe: the mass is lobulated and encapsulated, measuring 7.5 × 5.5 cm (**A**). The thymic tumor was not identifiable on gross examination (**B**).

**Fig. 3:**
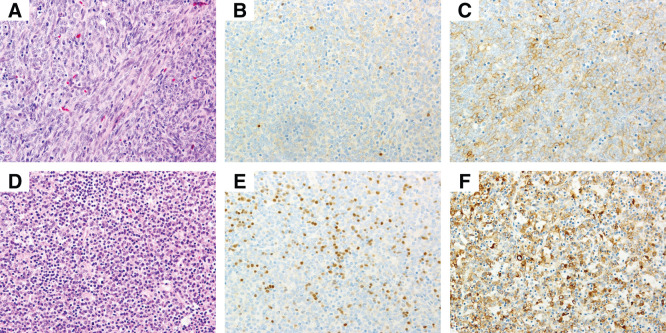
Pathological findings of the ectopic cervical thymoma. Upper panel: Type A region predominantly composed of proliferating spindle cells (**A**, H&E, original magnification ×20). TdT-positive immature T cells are scarce (**B**, TdT, original magnification ×20). Tumor cells showing weak positivity for pan-cytokeratin (**C**, AE1/AE3, original magnification ×20). Lower panel: Lymphocyte-rich type B region (**D**, H&E, original magnification ×20). TdT highlights scattered immature T lymphocytes (**E**, TdT, original magnification ×20). The tumor cells were positive for pan-cytokeratin (**F**, AE1/AE3, original magnification ×20). H&E, hematoxylin and eosin; TdT, terminal deoxynucleotidyl transferase

**Fig. 4:**
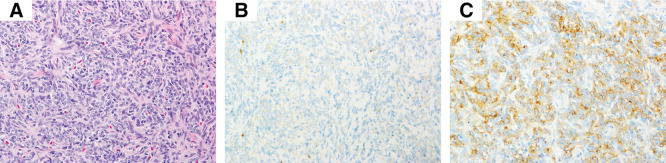
Pathological findings of the intrathymic thymoma. Intrathymic thymoma is composed predominantly of proliferating spindle cells (**A**, H&E, original magnification ×20). TdT-positive immature T cells are scarce (**B**, TdT, original magnification ×20). The tumor cells were positive for pan-cytokeratin (**C**, AE1/AE3, original magnification ×20). H&E, hematoxylin and eosin; TdT, terminal deoxynucleotidyl transferase

The ECT was lobulated, encapsulated, and measured 7.5 × 5.5 cm (**Fig. 2A**). In the extended thymectomy specimen, although not readily apparent on gross inspection (**Fig. 2B**), a 0.6-cm nodule was noted within the thymus (**Supplementary Fig. 2**). The cervical lesions consisted of a type A area predominantly composed of proliferating spindle cells and a lymphocyte-rich type B area. Although no invasion into the thyroid tissue was identified, invasion into the perithyroidal adipose tissue was confirmed (**Supplementary Fig. 3**). In the type A region, we identified sparse TdT-positive immature T cells, and the tumor cells showed weak immunoreactivity for pan-cytokeratin (**Fig. 3A**–**3C**, upper panel). The TdT-positive cells were sparse and clearly distinct from the cytokeratin-positive epithelial tumor cells. The lymphocyte-rich type B area contained abundant scattered immature T lymphocytes, as highlighted by TdT immunostaining, and the tumor cells were positive for pan-cytokeratin (**Fig. 3D**–**3F**, lower panel). The intrathymic lesion was predominantly composed of spindle cells with a few background lymphocytes. A fibrous capsule was present; however, focal extracapsular invasion was noted. Immunohistochemically, the tumor cells were positive for AE1/AE3; few immature TdT-positive T cells were identified, which were distinct from the epithelial tumor cells (**Fig. 4A**–**4C**). Therefore, the cervical lesion was diagnosed as a type AB ECT, and the intrathymic lesion was diagnosed as a type A thymoma (pT1aN0M0 Stage I, Masaoka stage II). Germinal centers were not identified in the thymic tissue.

The patient has remained recurrence-free as of 5 years postoperatively. The symptoms of MG have not substantially changed from before to after the operation, and the medication regimen has remained unchanged. In contrast, the dose of cyclosporine A used for managing PRCA has been reduced from 200 to 150 mg.

## DISCUSSION

Thymoma is a rare tumor arising from thymic epithelial cells, which accounts for less than 1% of all adult malignancies.^2)^ These tumors typically occur in the anterior mediastinum; however, various ectopic locations, such as the middle mediastinum,^3)^ neck, carotid triangle,^4)^ chest wall, pleura, lungs, and heart, have been reported.^2)^ Ectopic thymomas are thought to arise from residual thymic tissue located along the embryologic descent pathway of the thymus. These thymomas account for approximately 4% of all thymomas.^2,5)^

To date, 9 cases of ECT associated with MG have been reported (**Table 1**).^2,5–12)^ In addition, to our knowledge, no previous cases of ECT concomitant with both PRCA and MG have been reported, underscoring the singular rarity of the present case.

**Table 1:** Ectopic thymoma and myasthenia gravis

Author	Age	Sex	Tumor size (cm)	Surgery	Location	Clinical manifestations	WHO classification	Long-term MG outcomes
Zargar et al.^5)^	55	F	4.0 × 3.3 × 2.8	Tumor resection	Neck, inferior to the left thyroid lobe	Facial weakness, ptosis, dysconjugate eye movements, neck flexor weakness, dysphagia, and shortness of breath	A	Favorable
Mineo et al.^6)^	32	F	1.0 × 3.0	Tumor resection + ET	Neck, lower pole of the left thyroid gland	Shortness of breath, ptosis, myopathic facies, dysarthria, dysphagia, and generalized weakness	A	Favorable
Choi et al.^8)^	53	F	5.0 × 5.0 × 3.5	Tumor resection	Neck, left thyroid	Right-sided ptosis and weakness in both upper extremities	B1	Favorable
Kumazawa et al.^10)^	47	F	3.7 × 2.7	Tumor resection + ET	Neck, posterior to the right thyroid lobe	Ptosis, diplopia, fatigue, and mandibular weakness	B1	Favorable
Sato et al.^7)^	74	F	1.3 × 2.3	Tumor resection + ET	Neck, adjacent to the caudal thyroid	Fatigue and ptosis	B2	NA (AChR antibody 34.9 → 7.6)
Sekiguchi et al.^11)^	78	F	NA	Tumor resection	Neck	Dyspnea and shortness of breath	B2	Favorable
Kamimura et al.^12)^	61	F	4.5	Tumor resection + ET	Neck	Fatigue and ptosis	B2	Favorable
Marouf et al.^2)^	31	F	4.0 × 3.0 × 2.0	Tumor resection + ET	Neck, lower pole of the left thyroid gland	Ptosis, weakness, and rapid fatigue	AB	Favorable
Wu et al.^9)^	58	F	3.0 × 3.0 × 1.5	Tumor resection + ET	Neck, below the left thyroid lobe	Ptosis	AB	Favorable
Our case	65	F	7.5 × 5.5	Tumor resection + ET	Neck, within the left thyroid	Ptosis and diplopia	AB	No change (AChR antibody 3.7 → 2.2)

AChR, acetylcholine receptor; ET, extended thymectomy; MG, myasthenia gravis; NA, not available; WHO, World Health Organization

In thymoma-associated PRCA, the remission rate achieved by surgery alone is low, and immunosuppressive therapy with cyclosporine is the mainstay of treatment. Therefore, an extended thymectomy is not essential.^13)^ Accordingly, even in cases of ECT, tumor resection alone can be considered an acceptable therapeutic option. However, the optimal surgical approach for ECT associated with MG remains controversial. Among the 9 previously reported cases of ECT concomitant with MG, 6 patients underwent extended thymectomy, whereas the remaining 3 underwent simple excision of the ECT.

**Table 1** summarizes the 9 previously reported cases demonstrating the rare coexistence of ECT and MG. All reported patients, including our case, were female. Interestingly, in these 9 cases, the MG symptoms improved (at least minimally) after tumor resection, regardless of whether extended thymectomy was performed. This suggests the possibility of sparing extended thymectomy in patients with MG associated with ECT.^2,5–12)^

However, in the present case, in addition to the type AB ECT, an occult type A thymoma was identified in the mediastinal thymus. Few cases of thymoma arising in the thymus coexisting with an ectopic thymoma have been reported.^14,15)^ Considering the possibility that a thymoma undetectable on imaging may be hidden within the mediastinal thymus, as well as the potential involvement of the mediastinal thymic tissue itself in the pathogenesis of MG, the optimal strategy would be to resect both the thymus and the ectopic thymoma, provided the patient’s general condition permits this procedure.

Conversely, previous reports have shown that thymectomy may increase the long-term risk of developing other malignant tumors and may be associated with a poorer prognosis.^16)^ No other malignancy has been observed during the 5-year postoperative follow-up in the present case. However, a careful long-term surveillance remains necessary.

The incidence of multiple thymomas has been reported to be 1.1%–2.2% of all thymomas.^17)^ The type A thymoma within the thymus in the present patient may have been the lesion associated with MG and PRCA. However, Wang et al. analyzed 161 patients with thymoma and demonstrated that the incidence of MG was significantly higher in patients with type AB thymoma than that in type A (11.54% vs. 0.77%).^18)^ Regarding the histological distribution of thymoma-associated PRCA, Hirokawa et al. reported that among 17 cases in which the World Health Organization histological classification was available, type AB was the most frequent subtype (9 cases), whereas type A was observed in only 1 case (53% vs. 6%).^13)^ Therefore, in the present case, we presumed that MG and PRCA were associated with the type AB ECT rather than the occult type A thymic thymoma.

## CONCLUSIONS

Herein, we report a rare case of ECT associated with MG and PRCA. Further studies are warranted to clarify the relationship between ectopic thymoma, MG, and PRCA, and to determine the optimal treatment strategies.

## SUPPLEMENTARY MATERIALS

Supplementary Fig. 1 Port placement for video-assisted thoracoscopic extended thymectomy.

Supplementary Fig. 2 Gross findings of formalin-fixed postoperative specimens.

Supplementary Fig. 3 Histopathological evidence of the invasion of an ectopic thymoma into the perithyroidal thyroid tissue.

## DECLARATIONS

### Availability of data and materials

The patient-related data are not available for public access because of patient privacy concerns, but are available from the corresponding author upon reasonable request.
