# Supplementary material for: Ectopic Cervical Thymoma with Myasthenia Gravis and Pure Red Cell Aplasia: A Case Report
Source: Surg Case Rep. 2026 Mar 27;12(1):25-0818. doi: 10.70352/scrj.cr.25-0818 (PMC13043206; doi:10.70352/scrj.cr.25-0818)
Supplement: Supplementary Fig. 1 — Port placement for video-assisted thoracoscopic extended thymectomy. [file scr-12-01-25-0818-s001.pdf]

Supplementary Fig. 1 Port placement for video-assisted thoracoscopic extended thymectomy.

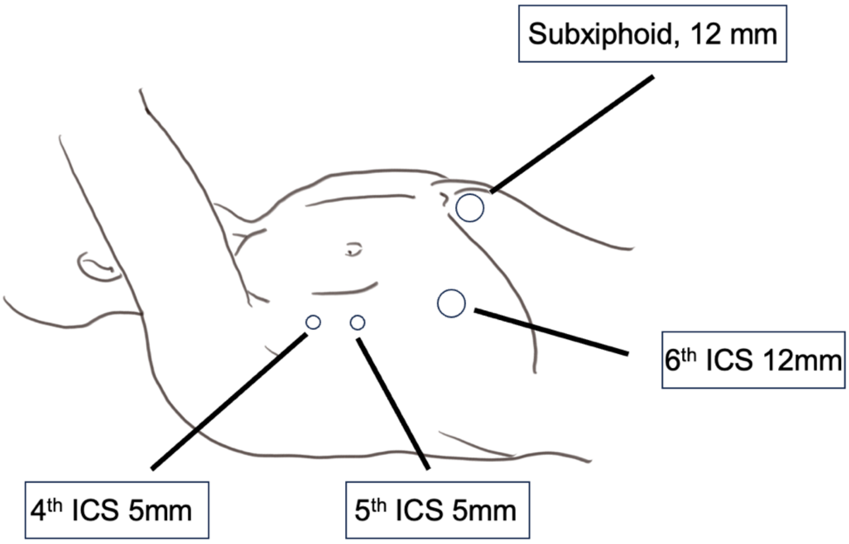

A schematic illustration of the four-port configuration, consisting of three right thoracic ports and one subxiphoid port.
ICS, intercostal space

Supplementary Fig. 2 Gross findings of formalin-fixed postoperative specimens.

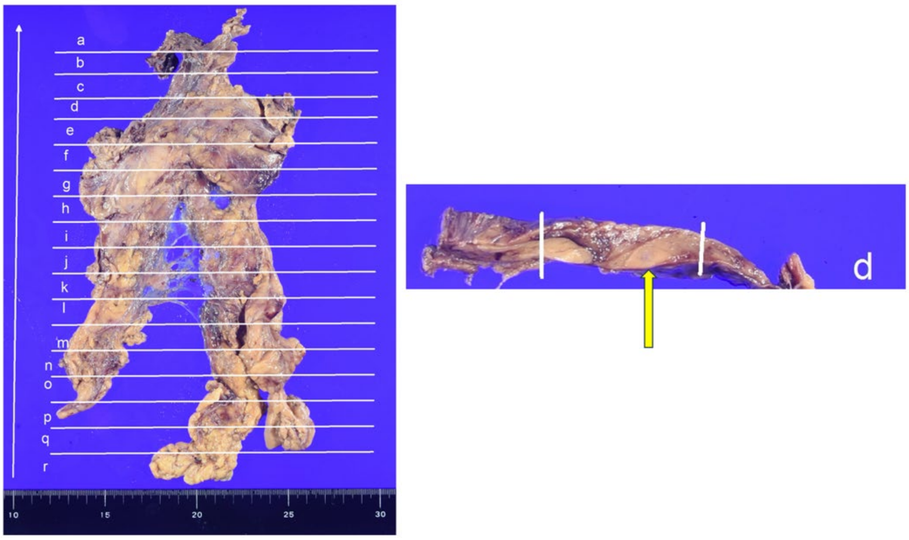

The tumor was not recognizable on the surface; however, a tiny nodule measuring 0.6 cm in diameter was identified on the cut surface (Magnified view of cut surface d, yellow arrow).

Supplementary Fig. 3 Histopathological evidence of the invasion of an ectopic thymoma into the perithyroidal thyroid tissue.

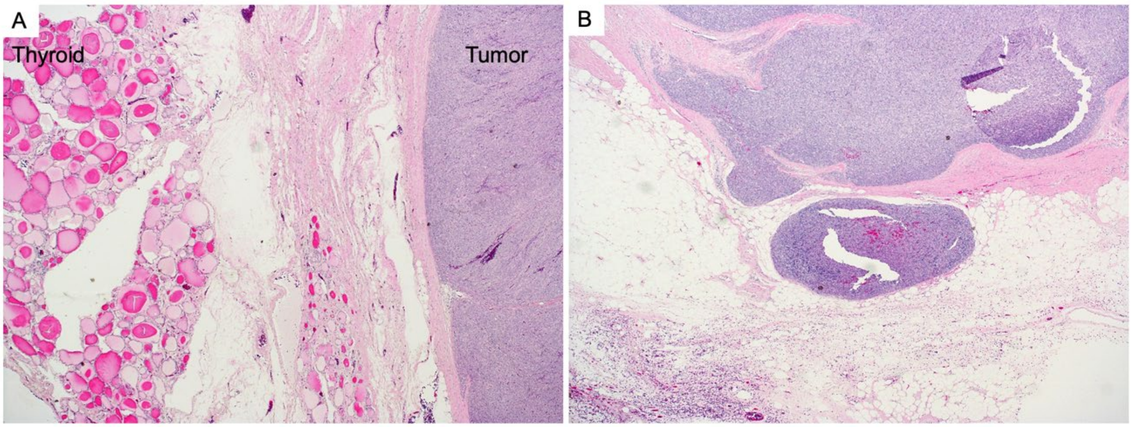

(A) The tumor showed no invasion into the thyroid parenchyma (original magnification ×20). (B) Invasion of the tumor into the perithyroidal adipose tissue was identified (original magnification ×20). Staining: hematoxylin and eosin.
